# Creating and disseminating a home-based cardiac rehabilitation program: experience from the Veterans Health Administration

**DOI:** 10.1186/s12872-019-1224-y

**Published:** 2019-11-06

**Authors:** Bonnie J. Wakefield, Kariann Drwal, Monica Paez, Sara Grover, Carrie Franciscus, Heather Schacht Reisinger, Peter J. Kaboli, Ramzi El Accaoui

**Affiliations:** 1 VA Office of Rural Health (ORH), Veterans Rural Health Resource Center-Central Region, Iowa City VA Healthcare System, 601 Highway 6 West, Mailstop 152, Iowa City, IA 52246-2208 USA; 2 The Comprehensive Access and Delivery Research and Evaluation (CADRE) Center at the Iowa City VA Healthcare System, 601 Highway 6 West, Mailstop 152, Iowa City, IA 52246-2208 USA; 3 The Department of Internal Medicine, University of Iowa Carver College of Medicine, Iowa City, USA

**Keywords:** Home based cardiac rehabilitation, Veterans affairs medical centers, Program implementation

## Abstract

**Background:**

Cardiac rehabilitation (CR) programs provide significant benefit for people with cardiovascular disease. Despite these benefits, such services are not universally available. We designed and evaluated a national home-based CR (HBCR) program in the Veterans Health Administration (VHA). The primary aim of the study was to examine barriers and facilitators associated with site-level implementation of HBCR*.*

**Methods:**

This study used a convergent parallel mixed-methods design with qualitative data to analyze the process of implementation, quantitative data to determine low and high uptake of the HBCR program, and the integration of the two to determine which facilitators and barriers were associated with adoption. Data were drawn from 16 VHA facilities, and included semi-structured interviews with multiple stakeholders, document analysis, and quantitative analysis of CR program attendance codes. Qualitative data were analyzed using the Consolidated Framework for Implementation Research codes including three years of document analysis and 22 interviews.

**Results:**

Comparing high and low uptake programs, readiness for implementation (leadership engagement, available resources, and access to knowledge and information), planning, and engaging champions and opinion leaders were key to success. High uptake sites were more likely to seek information from the external facilitator, compared to low uptake sites. There were few adaptations to the design of the program at individual sites.

**Conclusion:**

Consistent and supportive leadership, both clinical and administrative, are critical elements to getting HBCR programs up and running and sustaining programs over time. All sites in this study had external funding to develop their program, but high adopters both made better use of those resources and were able to leverage existing resources in the setting. These data will inform broader policy regarding use of HBCR services.

## Background

Cardiac rehabilitation (CR) programs provide significant benefit for people with cardiovascular disease [1–3]. Despite these benefits, such services are not universally available. Hospitals with limited resources, including many Veterans Affairs medical centers (VAMC) with a high percentage of rural patients, do not offer on-site CR programs. In the Veterans Health Administration (VHA), only 28% of VAMCs (35/124) have an on-site CR program. Of 47,051 CR-eligible VHA patients (i.e., hospitalized for myocardial infarction (MI), percutaneous coronary interventions (PCI), or coronary artery bypass graft (CABG) surgery in the VHA nationally from 2006 to 2011) only 8.4% participated in at least one session of CR in the 12-month post-hospitalization period. However, Veterans are significantly more likely to participate in CR if they are hospitalized at a facility with an on-site program (*p* < 0.001) [4]. VHA has established a mechanism to ensure veterans have access to CR through non-VHA care contracted services at private sector hospitals. However, even these contracted programs are not available in all areas and thus create a considerable travel burden for Veterans who live at distance from program sites. Veterans often have a co-pay for non-VHA programs, increasing their financial burden. Other barriers for Veterans, found also in the private sector [5, 6], include need for a driver because of comorbid conditions, time away from work, or being the primary caregiver to children or ill family members.

To address access issues and low attendance rates at on-site CR programs, investigators have evaluated home based CR (HBCR) programs. Differences in patient outcomes between home and on-site CR programs are minimal for risk factor modification, mortality, quality of life, clinical events, and costs [7–11]. However, unknown is whether patients, providers, and organizations would willingly adopt this approach in practice. For instance, anecdotally providers often cite patient safety concerns about use of HBCR programs.

**Fig. 1:**
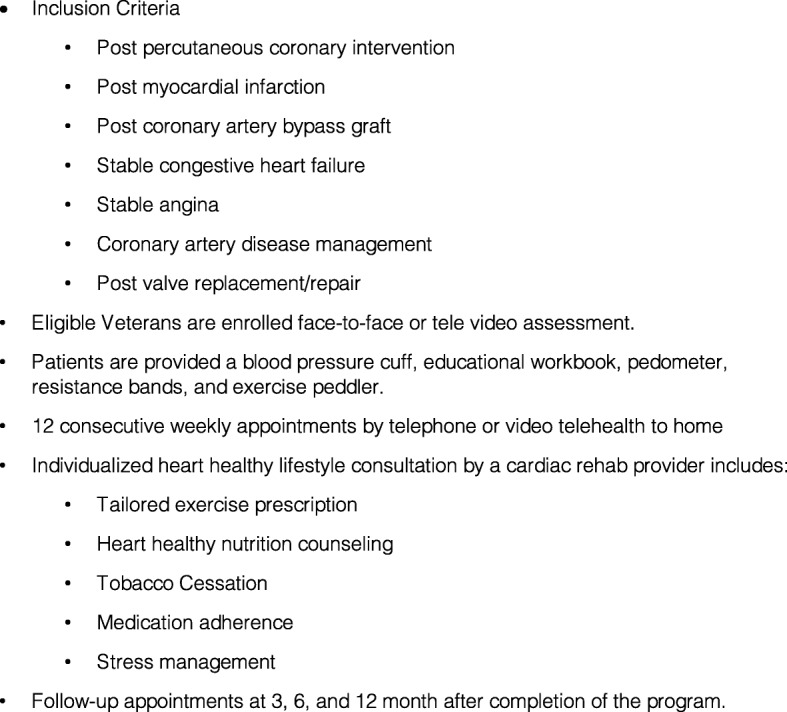
Components of the Home-Based Cardiac Rehabilitation Program

Given the evidence of the efficacy of CR, the effectiveness of HBCR programs, and access limitations to on-site programs, we developed, implemented, and evaluated a 12-week telephone/video-delivered HBCR program at the Iowa City Veterans Affairs Health Care System (ICVA). The program was structured to parallel on-site programs, spanning over 12 weeks. It provides patients with exercise prescription, education about their disease, medication compliance and heart healthy diet, assistance with tobacco cessation and psychosocial support. Program staff also relay any concerns or issues directly to the patient’s primary physician or cardiologist. Standardized clinic names and codes and note templates were developed in order to standardize documentation and facilitate retrieval of data for evaluation (Fig. 1).

In our initial evaluation, Veterans were offered a choice of HBCR or non-VHA center-based CR. More Veterans chose the HBCR program, patient outcomes were comparable in non-VA CR and HBCR, and a significantly greater number of sessions were completed in the HBCR arm. There were no reported adverse events in the HCBR population [8]. Following the initial pilot, an external facilitation approach has been used to disseminate the HBCR model to multiple VA sites across the country.

The primary aim of the study reported here was to examine factors associated with site-level implementation of HBCR. Specifically, we report barriers and facilitators associated with adoption of HBCR in high vs. low uptake HBCR programs.

## Methods

### Design

This study used a convergent parallel mixed-methods design [12] with qualitative data to analyze the process of implementation, quantitative data to determine low and high uptake of the HBCR program, and the integration of the two to determine which facilitators and barriers were associated with adoption. The ICVA and University of Iowa Institutional Review Board approved the study. Interview participants provided informed consent.

### Intervention

An external facilitation approach [13, 14] was used to disseminate the HBCR program to other VAMCs interested in implementing the model. Interested sites completed a detailed application to become a HBCR site. Each approved site received the HBCR tool kit materials, initial training, monthly mentoring calls, SharePoint site access, and ongoing consultation from the Iowa City VA HBCR staff.

### Study framework

We incorporated the Consolidated Framework for Implementation Research (CFIR) framework in this study [15] . The CFIR framework has been used in multiple implementation studies [16–18]. The framework includes 5 domains with associated constructs.

### Sample and data collection

Sixteen VAMCs participated in implementation over a 3-year period. We used documents from interaction with the sites to reflect the day-to-day implementation activities, barriers encountered, and discussion of potential solutions. Minutes were maintained for all monthly meetings, and all emails sent to or received by the external facilitator about the HBCR program were saved in electronic files [19].

To obtain a broader range of perceptions related to workflow processes, barriers, and facilitators to HBCR implementation at the organizational level, semi-structured telephone interviews were conducted with local VHA clinical leadership, providers, and program staff at a sample of nine geographically diverse HBCR sites across a range of uptake. An interview guide was developed using questions derived from the suggested interview questions provided by the CFIR wiki site (cfirguide.org). Questions addressed individual, organizational, cultural, and social factors that may influence support for HBCR. Site facilitators identified potential interviewees. Interviews lasted 8 to 92 min (average of 32).

To categorize high versus low uptake sites, patient participation data were obtained through the VHA Informatics and Computing Infrastructure (VINCI), including VHA inpatient and outpatient encounter files and Purchased Care files. National VHA files were used to identify all patients with an inpatient diagnosis of acute MI, undergoing PCI, or CABG surgery, the primary indications we used for the HBCR program. To assess CR participation following these qualifying events, we estimated the number of unique patients at each participating site who had an event in the first full fiscal year after the site entered into the study and who underwent one or more CR sessions at or paid for by the same site within 12 months after the qualifying event. Participation was categorized by 1) no participation, or attendance at one or more of the following: 2) VHA on-site CR programs; 3) non-VHA on-site CR programs; or 4) VHA home -based CR program.

### Data analysis

All textual data were uploaded in MAXQDA, a qualitative software program for data management and analysis. The unit of analysis was the organizational level. Qualitative data were analyzed using the CFIR coding framework modeled on prior evaluations. A priori, we made the decision to use all CFIR domains except *Domain IV. Characteristics of Individuals* as this implementation was focused on the organizational level. We added an additional category under External Setting (Networks & Communications, similar to Networks and Communication under Inner Setting) to describe communication occurring with outside organizations that may include other VHA facilities. Other modifications were made to the CFIR structure during the coding process. Under Inner Setting we created three sub-codes for this category: Program Characteristics, Organization Characteristics and Program Development. Under “External Change Agent” added “advice codes” to reflect advice sought and provided to implementation sites by the external facilitator. To ensure reliability in data coding, investigators coded the same emails/meeting minute transcripts and then met to examine the extent to which codes overlapped or diverged. The consensus approach was used to determine the final codes for analysis. Coding of interview transcripts was conducted in a similar fashion. Once initial coding was complete, data were then scored according to the influence of each CFIR category on implementation success [17, 20] using a modified scoring system where a score of 1 meant that the topic had a negative influence on implementation, 2 meant a neutral influence (neither positive nor negative), and 3 meant the topic had a positive influence.

## Results

The 16 HBCR implementation sites ranged from small rural facilities to urban tertiary care centers (Table 1). Overall, the team coded three years of documents, including 37 monthly meeting minutes and 743 email communications. For interview data, 22 telephone interviews were conducted with 10 local facilitators, 7 site physicians, and 5 local staff associated with the program (e.g., physical therapy manager) at 9 program sites. Combined this included 6205 individual coded segments: *n* = 2829 (46%) from emails and meeting minutes (excluding the advice segments); *n* = 1525 (25%) from interviews; and *n* = 1851 (30%) advice from external facilitator found in emails and meeting minutes.

**Table 1:** Participating Sites

Site ID	Overall Enrollment at site^1^	Qualifying Events^2^	Center Based (N)	% attend Center Based	VHA home-based with Qualifying Event (N)	% attend Home Based	Home Based, no documented Qualifying Event (N)
Low Adopter							
5	27,638	6	0	0.0%	0	0%	0
13	76,697	338	20	5.9%	0	0%	0
10	56,001	2	0	0.0%	0	0%	0
6^3,5^	85,253	215	11	5.1%	16	7%	28
8*	45,746	8	0	0.0%	0	0%	1
Medium Adopter							
11	62,210	211	21	10.0%	0	0%	26
18	84,196	128	14	10.9%	4	3%	13
1^4,5^	32,309	1	0	0.0%	0	0%	46
17*	116,980	143	0	0.0%	0	0%	30
High Adopter							
12^5^	46,163	257	5	1.9%	14	5%	54
16^5^	104,180	438	1	0.2%	19	4%	44
3	25,219	50	0	0.0%	5	10%	39
2^5^	73,404	178	2	1.1%	12	7%	60
9^5^	55,281	325	40	12.3%	94	29%	158
7^5^	169,809	485	1	0.2%	60	12%	228
4	62,463	393	148	37.7%	34	9%	47

^1^refers to the number of Veterans who have enrolled for care at each site

^2^# with myocardial infarction, percutaneous coronary intervention, coronary artery bypass graft; patients may have had care at more than one VA, so numbers are unique to each VA only; patients could also have had events/CR across multiple fiscal years and are counted only once per fiscal year; a qualifying event occurred no more than 3 months prior to the site’s start date; some patients had more than one qualifying event; CR must occur within 365 days after a qualifying event

^3^site 6 – site dropped out of program

^4^site 1 – outpatient only setting

^5^Interview sites

Facilities were grouped into high (*n* = 7), medium (*n* = 4), and low (*n* = 5) adoption facilities using natural cut points in the data (rate of HBCR participants following a qualifying event), HBCR participation (non- qualifying events). Table 1 indicates the number of patients at each site with qualifying events who attended center-based CR (VHA-based or private sector), and the number with qualifying events who attended HBCR. The last column in Table 1 indicates the number of patients who participated in HBCR for whom no qualifying event was documented in the national data base, e.g., patients with stable heart failure could be referred to the program. Qualifying events were determined for each site, however patients may have had care at more than one VA, so numbers are unique to each VA only. A qualifying event was counted if it occurred no more than 3 months prior to the site’s start date; CR occurring up to 365 days after a qualifying event were included. We defined “adoption” as continued participation in the program past the first year [17]*.* We used the influence ratings to provide an estimate of the magnitude (positive or negative) of each construct. Only high and low uptake sites are included in this analysis.

The prominent difference between high and low uptake facilities influence scores was in Readiness for Implementation, which includes leadership engagement, available resources, and access to knowledge and information and Process factors, including planning, champions and opinion leaders (Table 2).

**Table 2:** Differences between High and Low Adoption Programs^1^

	High Adopters Mean (SD) ^2^	Low Adopters Mean (SD) ^2^
INNER SETTING		
Readiness for implementation:		
Leadership engagement	2.2 (0,7)	1.4 (0.3)
Available resources	2.4 (0.3)	1.7 (0.6)
Access to knowledge	3 (0)	2.1 (0.5)
PROCESS		
Planning	2.9 (0.1)	2.3 (0.2)
Engaging: Champions	2.8 (0.2)	2 (0.7)
Engaging: Opinion leaders	2.8 (0.2)	2 (1.0)

^1^All differences at *p*-value ≤0.05

^2^1 = negative influence; 2 = neutral influence; 3 = positive influence

### Leadership engagement

In the high uptake organizations, clinical leadership was evident. Although local clinical leadership was strong in high uptake facilities, less support was displayed in organizational leadership roles. In contrast, low uptake sites struggled obtaining leadership support.

### Available resources

In the high uptake group, the ability to get staff up and going efficiently facilitated implementation of the program. These facilities also had local organizational resources and collaborations available to complement program activities, such as active physical therapy departments. High uptake sites did encounter barriers with resources, e.g., finding space to conduct the program, but seemed to be able to address the barrier compared to low uptake sites. In fact, one site found the home-based program provided a solution to the lack of on-site space for a center based program. In contrast to the high uptake sites, low uptake sites seemed to have fewer organizational resources.

### Access to knowledge and information

High uptake sites were able to take advantage of the knowledge provided by the program SharePoint and external facilitator staff. Low uptake sites had less information at the local level, and when information was available (as on the SharePoint site), using it presented challenges.

However, low uptake sites did express the helpfulness of the provided information, both from the initial training and information on the SharePoint.

Process factors, including Planning, champions and opinion leaders were also more prevalent at high adoption sites. High adoption sites engaged in active program planning. Although low adopters did spend time planning, the process was often much slower at these sites. Opinion leaders at high adopter sites were more engaged with the process of implementation. Low adopter sites encountered difficulty engaging opinion leaders.

Finally, high adopter sites typically had local champions to help move implementation forward. At high adoption sites, support from providers, particularly local cardiologists, was key in success. However, low adoption sites often exhibited changes in leadership (both clinical and administrative) that led to a lack of champions. For example, one site had a very supportive Chief of Staff, who initially championed the program. However, after he left, support was not provided.

The frequency of Advice codes was also different between high and low uptake sites. In the high uptake sites, requests for advice ranged from 18 to 35 per year (average 24/site/year). In low uptake sites, requests ranged from 9 to 24 per year (average 16/site/year).

## Discussion

Comparing high and low uptake programs, readiness for implementation (leadership engagement, available resources, and access to knowledge and information), planning, and engaging champions and opinion leaders were key. Analysis of the external facilitator’s emails also indicated that advice was frequently needed on funding, structural characteristics, using available resources, and access to knowledge and information. High uptake sites were also more likely to seek information from the facilitator, compared to low uptake sites.

There were few adaptations to the design of the program at individual sites. All sites used the same electronic medical record and patient program materials. The most common adaptation was the profession of the local site facilitator, e.g., although the external facilitator was an exercise physiologist, some sites chose to use registered nurses or physical therapists. Site facilitators and interview participants were very positive about the design of the program, the helpfulness of the external facilitator, and acknowledged that the program met patient needs for access to care. Because the VHA uses one EMR, sharing note templates and clinic codes across all sites facilitated standardized data entry formats and accessing clinic data for program uptake estimates. Thus, standardized data entry approaches and common clinic codes across the country will facilitate evaluation of data contained in the database.

Successful sites demonstrated both strong administrative and clinical leadership, particularly from cardiologists involved in program implementation. These physicians championed the program and communicated with hospital leadership about the importance of the program. Low uptake programs often exhibited inconsistent leadership, i.e., frequent changes in leadership, departure of a supportive physician leader who was then replaced by a less supportive or non-supportive leader. One low adopter site spent the first 10 months in planning activities, and had difficulty implementing the program.

High uptake sites appeared to both have greater available resources and were better able to leverage local resources to facilitate program implementation. Successful sites often used existing support staff such as nutritionists, and took advantage of available technology, i.e., secure messaging, to improve efficiency of weekly visits. While space was a potential barrier, high uptake sites developed workarounds to address the space problem. For example, at one site, the lack of space was addressed by allowing HBCR providers to work from home (telework) to implement the program.

All sites had access to the SharePoint site for program toolkit materials and were invited to on-site training. While all programs found these sources of information very helpful, low uptake sites had greater difficulty taking advantage of this information. Furthermore, high uptake sites were more likely to seek out additional sources of information, such as professional society websites, to augment program materials, as well as advice and information from the external facilitator.

Since these data were collected, the national program has grown to 28 sites, including 9 spoke sites. Hub sites, typically larger sites, provide HBCR program services to patients enrolled at mostly smaller, rural (spoke) sites who do not have a large population of patients for a full HBCR program. A recent analysis of CR within the VHA found that Veterans hospitalized at a facility with a HBCR program were 3 to 4 times more likely to participate in HBCR relative to an on-site VA program or non-VA program respectively [21]. Our initial program evaluation found that Veterans preferred the HBCR program to on-site non-VA programs [8]. Further implementation is supported by national guidelines that recommend use of HBCR for low risk patients [9]. Unfortunately, participation rates in either center-based or HBCR in this population remained low, a well-documented problem in CR programs [22, 23].

There are limitations to the study. The study was conducted within VHA facilities limiting external validity. Outside the VHA, differences in electronic records, human resources, finance, organizational climate, and processes will require “ground up” design and implementation. We do not report clinical outcomes, including adverse events. Grouping of sites by adoption levels may be viewed as arbitrary. Document analysis data were not created for purposes of research and thus are not as rigorous as interviews or focus groups, and important factors may go unmentioned. Although HBCR is more accessible, our program had low referral and enrollment rates, which is a long-standing issue in cardiac rehabilitation programs [24–26]. Lastly, a major barrier to adoption of HBCR in the United States is lack of insurance coverage for this type of home-based program.

## Conclusions

Understanding how each VAMC implemented HBCR in their network, the role of facilitation in guiding program development, and local adaptations to program operation provided insight into initial and sustained acceptance of HBCR among a variety of stakeholders. Like other implementation efforts [27, 28] consistent and supportive leadership, both clinical and administrative, are critical elements to getting programs up and running and sustaining programs over time. All sites in this study had external funding to develop their program, but high adopters both made better use of those resources and were able to leverage existing resources in the setting. Although we did not test this association, it is likely that leadership and available resources are linked to each other, leading to successful implementation.

Most program implementation in health care settings is driven by a “top-down” (push) approach. This study adds to the implementation literature by examining factors associated with implementation using a “bottom-up” (pull) approach. Thus, these data provide critical information for healthcare institutions to begin to close the gap in access to CR services for patients regardless of where they reside. These data will also inform broader policy regarding use of HBCR services.

## Data Availability

The datasets used and/or analyzed during the current study are available from the corresponding author on reasonable request.

## Abbreviations

CABG: Coronary artery bypass graft surgery
CFIR: Consolidated framework for implementation research
CR: Cardiac rehabilitation
HBCR: Home-based cardiac rehabilitation
ICVA: Iowa City veterans affairs health care system
MI: Myocardial infarction
PCI: Percutaneous coronary interventions
VAMC: Veterans affairs medical centers
VHA: Veterans health administration
VINCI: VHA informatics and computing infrastructure
